# Transcriptome–Proteome Analysis of Human Naive and Memory B Cell Subsets Reveals Isotype and Subclass‐Specific Phenotypes

**DOI:** 10.1002/eji.70159

**Published:** 2026-03-12

**Authors:** Jana Koers, Arie J. Hoogendijk, Simon Tol, Floris P.J. Van Alphen, Ninotska I.L. Derksen, Maartje van den Biggelaar, Theo Rispens

**Affiliations:** ^1^ Sanquin Research and Landsteiner Laboratory Amsterdam University Medical Centers University of Amsterdam Amsterdam The Netherlands; ^2^ Amsterdam UMC location Vrije Universiteit Amsterdam Molecular Cell Biology and Immunology Amsterdam The Netherlands; ^3^ Amsterdam institute for Immunology and Infectious Diseases Immunology Amsterdam The Netherlands

**Keywords:** B cells, IgG4, Isotypes, proteomics, transcriptomics

## Abstract

Antibodies produced by B cells aid in the recognition and clearance of pathogens and are the cornerstone of vaccination strategies. Humans produce nine different antibody isotypes, and their effector functions differ according to the type of antigen and route of exposure. Phenotypic variation between isotype‐switched B cell subsets is expected but not studied in detail. To obtain a molecular definition of isotype‐defined cell identity, we performed proteomics and transcriptomics on isotype‐defined populations of human naive and memory B cells (MBCs): CD27^−^IgM^+^IgD^+^, CD27^+^CD38^lo/−^IgM^+^IgD^+^, CD27^+^CD38^lo/−^IgM^+^IgD^−^, and IgA1, IgA2, IgG1, IgG2, IgG3, and IgG4 MBCs (CD27^+^CD38^lo/−^Ig^+^). Combined proteome and transcriptome analysis revealed that mRNA and protein expression profiles separate isotype‐defined B cell subsets according to their differentiation status. mRNA and protein expression levels correlated reasonably well for many genes. IgG4‐switched B cells were most distinct from naive B cells in terms of mRNA as well as protein expression profiles. Besides a distinct expression profile of cytokine and Fc receptors, we identified a high expression of IgE‐coding mRNA in IgG4‐switched B cells. SDR16C5 was identified as uniquely upregulated in IgG4‐switched B cells. Taken together, this study highlights the distinct phenotypic profile of IgG4‐switched B cells.

## Graphical abstract

We performed combined proteome and transcriptome analysis of human isotype‐defined B cell subsets.

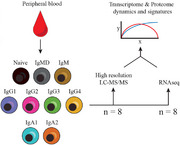

## 1 Introduction

The ability of B cells to produce antibodies is essential for effective humoral immunity by recognition and clearance of pathogens. During B‐cell maturation, immunoglobulin (Ig) genes are randomly rearranged to generate B‐cell receptors (BCRs), which are expressed on the B‐cell surface. BCRs exist in different isotypes, including IgD, IgM, IgG, IgA, and IgE. For IgG, four subclasses are described (IgG1, 2, 3, and 4) and two for IgA (IgA1 and 2). Antigen‐naive B cells co‐express IgM and IgD BCRs. Upon antigen encounters, B cells are able to express other antibody isotypes via a process known as class‐switch recombination. For effective humoral responses, the expression of the appropriate isotype is essential. The route of antigen exposure and the context in which B cell activation occurs govern selection for a specific isotype, which in turn can influence B cell fate and function [1, 2, 3].

Activated naive B cells participate in either extrafollicular or germinal center (GC) responses, and formation of isotype‐switched B cells occurs in both [4, 5, 6, 7, 8]. Memory B cells (MBCs) can be generated before the onset of GCs, or at early and late stages during a GC reaction [9, 10, 11, 12, 13]. MBCs aid in the generation of immunological memory essential for rapid and effective response to previously encountered pathogens and their related variants [14] and are the cornerstone of vaccination. MBCs are long‐lived and recirculate through the periphery or take up residence in tissues in a quiescent state. Upon secondary antigen exposure, MBCs can undergo further diversification by re‐entry into the GC reaction with subsequent isotype‐switching, or differentiate into antibody‐secreting cells in a GC‐independent fashion [15, 16, 17, 18]. B cell fate decision is among others influenced by the expressed isotype and function of MBCs, as well as antibody‐secreting cells is dependent on the expressed isotype [16, 17, 19, 20]. Hence, control of isotype expression is relevant to the outcome of both primary and secondary responses.

Naive B cells that predominantly express IgD are anergic, and the function of IgD antibodies is still incompletely understood and represents the second least abundant isotype in serum [21, 22]. IgM is the first antibody to be expressed in the early stages of an immune response in anticipation of affinity‐matured antibodies such as IgG and IgA. IgG antibodies are most abundant in serum and confer a central role in systemic immunity, and they are often the primary effector antibody raised in response to inflammation, whereas IgA fulfills essential roles in the mucosal immune system. IgE^+^ B cells are extremely rare in peripheral blood, and IgE antibodies mediate allergic reactions and confer powerful effector functions via FcεRs.

Of the four IgG subclasses, IgG4 stands out as it is typically only a minor fraction of an antibody response against bacteria or viruses [23, 24]. However, prolonged and/or repeated antigen challenge in the absence of infection can result in a highly IgG4‐skewed antibody response, such as during allergen‐specific immunotherapy. In this setting, IgG4 titers correlate with relief of allergic symptoms. However, many autoimmune diseases are also characterized by IgG4 autoantibodies. Therefore, possibilities for selective targeting of IgG4‐switched B cells may be therapeutically important.

Phenotypic data on B cells expressing different antibody subclasses are sparse, and whether isotype specificity is also reflected at the cellular level is unclear. Phenotyping studies using flow cytometry previously performed in our lab and by others revealed differences between B cells expressing different IgG subclasses [25, 26, 27, 28]. IgG4 B cells were found to have reduced expression of chemokine receptor CXCR4 and CXCR5 and complement receptor 2 (CR2/CD21), but higher expression of IgεRII/CD23, compared with IgG1 B cells. Furthermore, IgG4 does not express CCR7, restricting its entry into secondary lymphoid organs and thus most frequently resides within the peripheral blood. To obtain a comprehensive and unsupervised molecular definition of isotype and subclass‐defined B cell identity, we performed proteomics and transcriptomics for quantitative comparison of human naive and isotype and subclass‐defined memory B cells (MBCs).

## 2 Methods

### 2.1 B Cell Isolation and Cell‐Sorting

Healthy adult human peripheral blood was obtained with written informed consent in accordance with the guidelines established by the Sanquin Medical Ethical Committee and in line with the Declaration of Helsinki. Donor information is summarized in Table S1. Blood samples were drawn outside of the seasonal allergy season. Peripheral blood mononucleated cells (PBMCs) were isolated from fresh buffy coats using Ficoll gradient centrifugation (Lymphoprep; Axis‐Shield PoC AS). PBMC fractions were screened for adequate yet normal IgG4 MBC frequencies (0.2%–1% of total CD19^+^). Total B cells were isolated by untouched magnetic bead separation (Pan B cell isolation kit human; Miltenyi Biotec), and the studied subsets were separated using flow cytometric cell‐sorting based on expression of CD19, CD27, CD38, and BCR isotype (FACS Aria III and FACS Aria IIIu, BD Biosciences) [29]. During sorting, cells that expressed multiple BCR isotypes, due to promiscuous antibody staining or recent class switch events, were excluded.

### 2.2 Antibodies

The following antibodies were included: anti‐CD19 (clone SJ25C1, 563325), anti‐IgD (clone IA6‐2, 561315), and anti‐IgM (clone G20‐127, 562618) from BD Biosciences. Anti‐IgA1 (clone B3506B4, 9130‐30), anti‐IgA2 (clone A9604D2, 9140‐31), and anti‐IgG2 (clone HP6002, 9070‐02) from SouthernBiotech. Anti‐IgG1 (clone HP6188, M1325), Anti‐IgG3 (clone HP6095, M1270), and anti‐IgG4 (clone HP6098, M1271) from Sanquin Reagents. Anti‐CD27 (clone O323, 63‐0279‐42), anti‐CD38 (clone HB7, 25‐0388‐42), and Live/Dead Fixable Near‐IR Dead cell stain (L10119) from ThermoFisher Scientific. Each antibody was titrated to optimal staining concentration using PBMCs.

### 2.3 Sample Preparation for Mass Spectrometry

FACS‐separated B cell subsets from eight donors were sorted into Protein LoBind tubes (Eppendorf) and washed with PBS. For IgG4, four donors were pooled to reach sufficient cell numbers for proteomics analysis (*n* = 4), and for IgG3, two donors were pooled (*n* = 6) (Table S2). Tryptic peptides were prepared similarly to a previously described method [30]. Briefly, depending on the acquired cell number per sample (Table S2) cells were lysed in 10–25 uL 1% sodium deoxy cholate (SDC) (Sigma Aldrich, Germany) 10 mM TCEP (Thermo Scientific, USA), 40 mM ChloroAcetamide (Sigma Aldrich), 100 mM TRIS‐HCl pH 8.0 (Life Technologies, UK), boiled at 95°C for 5 min and sonicated for 10 min in a BioRuptor Pico (Diagenode, Belgium). A double volume of 100 mM TRIS‐HCl pH 8.0 was added, containing 125–325 ng Trypsin/LysC (Thermo). Samples were digested overnight at 25°C. Next, samples were acidified by the addition of 1% (v/v) trifluoroacetic acid (Thermo Scientific), centrifuged, and supernatants containing the peptides were loaded on in‐house prepared SDB‐RPS STAGEtips (Empore, USA). STAGEtips were washed with 0.1% TFA, and peptides were eluted in 5% (v/v) ammonium hydroxide (Sigma Aldrich), 80% v/v acetonitrile (BioSolve). Sample volume was reduced by SpeedVac and supplemented with 2% acetonitrile, 0.1% TFA to a final volume of 10 µL; 3 µL of each sample was injected for MS analysis.

### 2.4 Mass Spectrometry Data Acquisition

Tryptic peptides were separated by nanoscale C18 reverse phase chromatography coupled on line to an Orbitrap Fusion Tribrid mass spectrometer (Thermo Scientific) via a nanoelectrospray ion source (Nanospray Flex Ion Source, Thermo Scientific). Peptides were loaded on a 20 cm 75–360 µm inner–outer diameter fused silica emitter (New Objective) packed in‐house with ReproSil‐Pur C18‐AQ, 1.9 µm resin (Dr. Maisch GmbH). The column was installed on a Dionex Ultimate3000 RSLC nanoSystem (Thermo Scientific) using a MicroTee union formatted for 360 µm outer diameter columns (IDEX) and a liquid junction. The spray voltage was set to 2.15 kV. Buffer A was composed of 0.1% formic acid, and buffer B of 0.1% formic acid, 80% acetonitrile. Peptides were loaded for 17 min at 300 nL/min at 5% buffer B, equilibrated for 5 min at 5% buffer B (17–22 min) and eluted by increasing buffer B from 5–15% (22–87 min) and 15–38% (87–147 min), followed by a 10 min wash to 90% and a 5 min regeneration to 5%. Survey scans of peptide precursors from 400 to 1500 m/z were performed at 120K resolution (at 200 m/z) with a 4 × 105 ion count target. Tandem mass spectrometry was performed by isolation with the quadrupole with an isolation window of 1.6, HCD fragmentation with a normalized collision energy of 30, and rapid scan mass spectrometry analysis in the ion trap. The MS2 ion count target was set to 1.5 × 104 and the max injection time was 35 ms. Only those precursors with charge state 2–7 were sampled for MS2. The dynamic exclusion duration was set to 60 s with a 10 ppm tolerance around the selected precursor and its isotopes. Monoisotopic precursor selection was turned on. The instrument was run in top speed mode with 3 s cycles.

### 2.5 RNA Extraction

FACS‐separated B cell subsets from eight donors were sorted into DNA LoBind tubes (Eppendorf) and washed with PBS. Total RNA was isolated from B cell sorted subsets using Qiagen RNeasy Plus Universal mini kit according to the manufacturer's instructions (Qiagen, Hilden, Germany). RNA samples were quantified using Qubit 4.0 Fluorometer (Life Technologies, Carlsbad, CA, USA), and RNA integrity was checked with RNA Kit on Agilent 5300 Fragment Analyzer (Agilent Technologies, Palo Alto, CA, USA). Total RNA samples that had inadequate quantity or an RNA integrity number (RIN) < 8 were excluded from the library preparation.

### 2.6 RNA Library Preparation and Sequencing

RNA sequencing library preparation was prepared using NEBNext Ultra II Directional RNA Library Prep Kit for Illumina, following the manufacturer's instructions (NEB, Ipswich, MA, USA). Briefly, mRNAs were first enriched with Oligo(dT) beads. Enriched mRNAs were fragmented. First‐strand and second‐strand cDNA were subsequently synthesized. The second strand of cDNA was marked by incorporating dUTP during the synthesis. cDNA fragments were adenylated at 3′ends, and an indexed adapter was ligated to the cDNA fragments. Limited‐cycle PCR was used for library amplification. The dUTP incorporated into the cDNA of the second strand enabled its specific degradation to maintain strand specificity. Sequencing libraries were validated using the NGS Kit on the Agilent 5300 Fragment Analyzer (Agilent Technologies) and quantified by using Qubit 4.0 Fluorometer (Invitrogen, Carlsbad, CA, USA). The sequencing libraries were multiplexed and loaded on the flowcell on the Illumina NovaSeq 6000 instrument according to the manufacturer's instructions. The samples were sequenced using a 2 × 150 pair‐end (PE) configuration v1.5. Image analysis and base calling were conducted by the NovaSeq Control Software v1.7 on the NovaSeq instrument. Raw sequence data (.bcl files) generated from Illumina NovaSeq were converted into fastq files and de‐multiplexed using the Illumina bcl2fastq program version 2.20. One mismatch was allowed for index sequence identification.

### 2.7 Data Processing and Analysis

Raw MS data files were acquired with XCalibur software (Thermo Fisher Scientific) and processed with MaxQuant 1.6.2.10 software [31]. Peptides were searched against the homo sapiens Uniprot database (downloaded March 2019, 73,932 entries). Enzyme specificity was set to trypsin with a maximum of 2 missed cleavages, and carbamidomethylation on cysteine residues was used as a fixed modification. The false discovery rate (FDR) was 0.01 for peptide and protein levels with a minimum length of 7 amino acids. Label‐free quantitation (LFQ) was performed with a minimum ratio count of 2 based on unique peptides for quantification. MaxQuant output files were loaded in R 4.1.2 for further analysis. Proteins were filtered by “potential contaminant”, “reverse”, and “only identified by side”, and LFQ intensities were log2 transformed. Statistical significance was determined with moderated t‐tests using the limma package [32]. A Benjamini‐Hochberg multiple testing corrected *p*‐value <0.05 and an absolute log_2_ fold change of >1 was considered statistically significant and relevant. RNA fastq sequencer output quality was determined using FastQC, and transcripts were aligned to the human hg38 genomic reference (release 104) sequence using STAR [33]. Differential expression analysis was performed using DESeq2 [34], applying a significance threshold of a Benjamini‐Hochberg multiple testing corrected *p*‐value of <0.05 and an absolute log2 fold change of >1. Correlation between data and disorder‐specific theoretical protein profiles was performed using Pearson correlation. Transcriptomes and proteomes were merged based on ENSEMBL identifiers, and co‐expression analysis was performed using the WGCNA package [35]. Gene ontology and pathway overrepresentation analyses were performed using the clusterprofiler package [36].

### 2.8 Statistical Analysis

Differences between groups were analyzed using a one‐way ANOVA and Tukey's multiple comparison test (each group against every other group). A *p*‐value <0.05 was considered significant. Statistical tests, excluding those evaluating RNAseq and proteomics data, were performed using GraphPad Prism 9.1.1.

## 3 Results

### 3.1 Transcriptomics and Proteomics of Human Peripheral Blood Naive and Subclass‐Defined Memory B Cells

**FIGURE 1:**
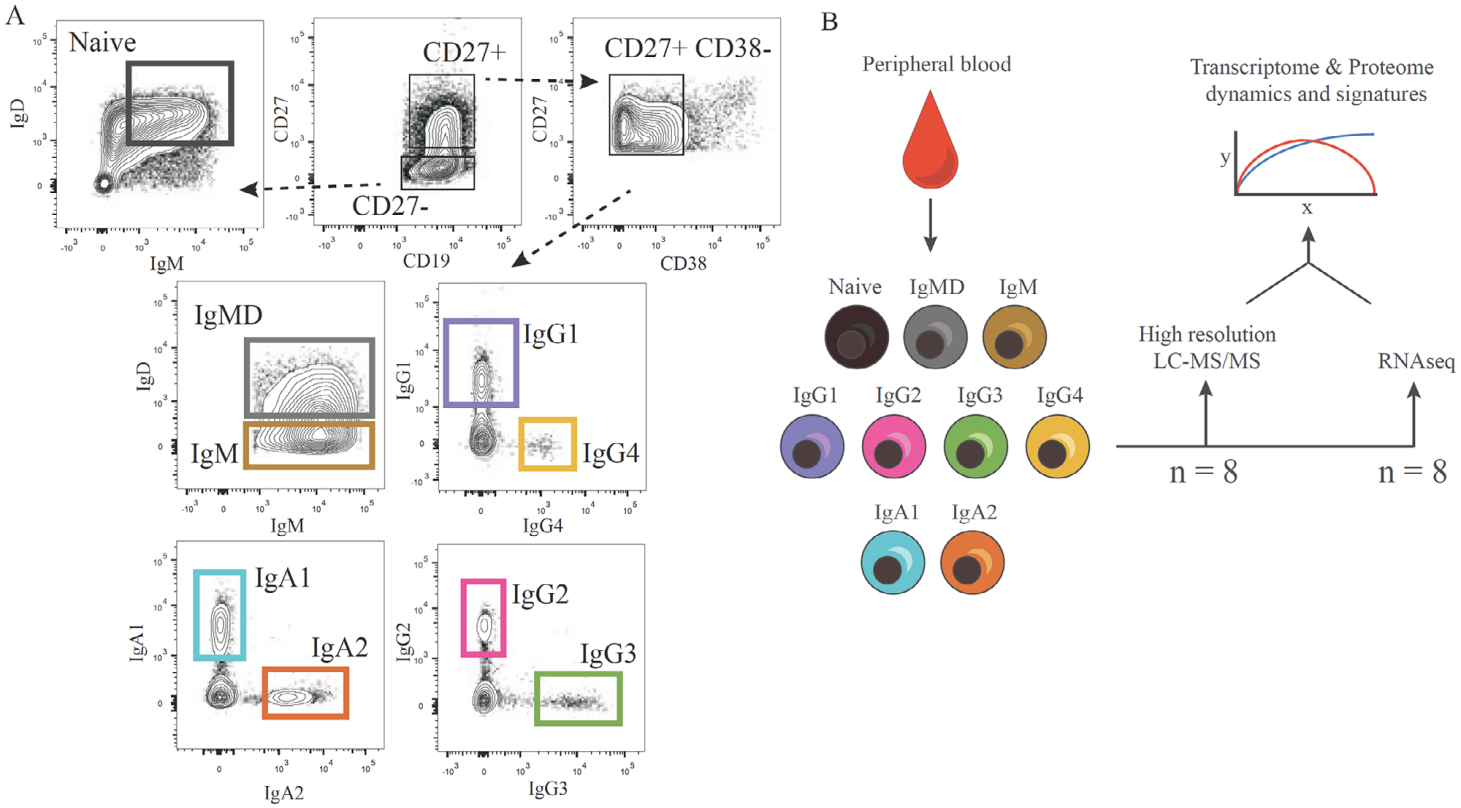
Transcriptomics and Proteomics of human peripheral blood naive B cells and Ig‐isotype defined memory B cells. (**A**) Representative flow cytometric profiles of isolated human B cell subsets. For more details, see Figure S1. (**B**) Schematic representation of experimental set‐up and analysis. Naive B cells and memory B cell (MBC) subsets were isolated by gradient centrifugation and flow cytometry and analyzed by liquid chromatography‐tandem mass spectrometry (MS) and RNA sequencing.

To define subclass‐defined MBC identity at the molecular level, we performed quantitative proteomics and transcriptomics (Figure 1; Figure S1). To this end, nine CD19^+^ B cell subsets were isolated from peripheral blood mononuclear cells (PBMCs) of healthy donors (*n* = 16). These subsets comprised naive B cells (CD27^−^IgM^+^IgD^+^), nonswitched MBCs (CD27^+^ CD38^lo/−^IgM^+^IgD^+^), IgM MBCs (CD27^+^ CD38^lo/−^IgM^+^IgD^−^), and isotype switched IgA1, IgA2, IgG1, IgG2, IgG3, and IgG4 MBCs (CD27^+^ CD38^lo/−^ IgA1‐2^+^/IgG1‐4^+^; Figure 1A). CD27^+^ B cells that express IgE BCRs are extremely rare in human peripheral blood [37] and were therefore not included in this study. Due to the low frequency of IgG4 B cells, we did not further segregate memory B cell subsets using additional phenotypic markers [38]. RNA sequencing on the nine B cell subsets (*n* = 8) identified a total of 60,664 unique mRNAs. High‐resolution mass spectrometry (MS) identified 3511 proteins across all subsets (*n* = 8, Figure S2B), in line with a similar proteome analysis on CD4 T cell subsets [39]. To evaluate the purity of the sorted B cell populations, we assessed the expression levels of the different immunoglobulin isotypes at both the transcriptomic and proteomic levels within each subset. As illustrated in Figure S2, the predominant immunoglobulin isotype detected in each dataset corresponds to the isotype used for sorting, confirming that the sorted populations are highly enriched for the intended subsets. We have also used stringent gating for the isotype‐enriched subsets, as can be seen from the sorting strategy PDFs. It should be noted, however, that due to potential class switch recombination (CSR), some transcripts corresponding to other Ig isotypes can be detected. As these analyses were performed on bulk‐sorted populations, the resulting expression profiles represent the average signal of all cells within a subset rather than single‐cell resolution. Therefore, minor expression of alternative isotypes is expected and consistent with known biological processes of B cell differentiation.

### 3.2 B Cell Differentiation According to Isotype Identity

**FIGURE 2:**
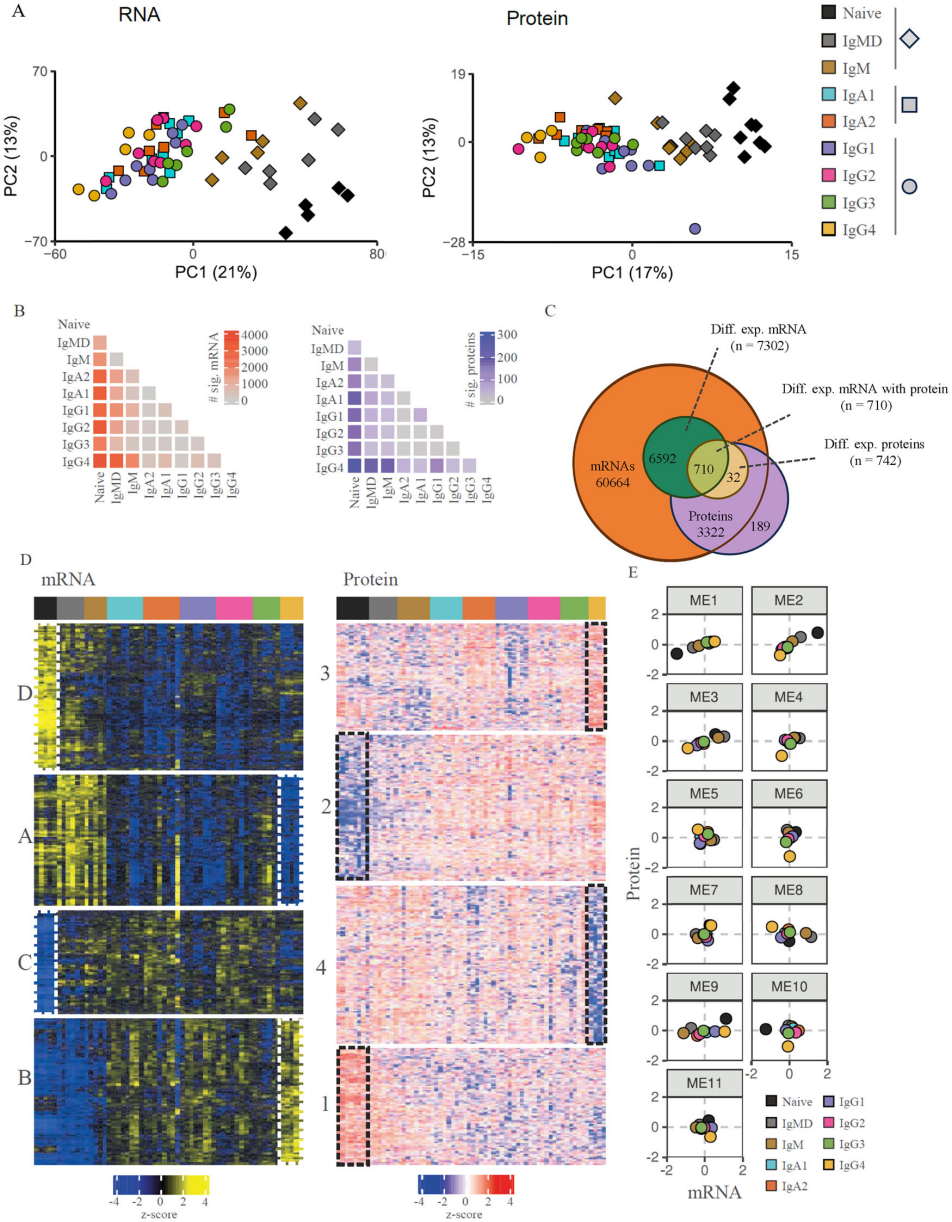
Transcriptomics and proteomics profiles of B cell subsets. (**A**) Principle component analysis (PCA) plot of transcriptomes (left) and proteomes (right). Each color represents a subset, and each symbol a single donor. (**B**) Heatmaps display the number of significantly differently expressed mRNAs (left) or proteins (right). (**C**) Venn diagram showing expressed mRNA, differentially expressed mRNA, quantified proteins, and differentially expressed proteins. Note that for most proteins, we detected mRNAs, but not vice versa. (**D**) K‐means clustering showing relative mRNA (left) and protein (right) expression levels (z‐score) of the differentially expressed genes (*n* = 7308, *p* < 0.05) and proteins (*n* = 738, FDR < 0.05) among B cell subsets (subsets imposed; see Figure S5 for hierarchical clustering). Colored columns respond to different subsets (*n* = 4–8; 8 donors for proteomics and 8 donors for transcriptomics, in some cases, subsets from several donors were pooled, see Table S2). Letters (mRNA) and numbers (protein) indicate clusters. Dashed white (mRNA) or black (protein) lines represent distinct expression signatures for naive B cells and IgG4 MBCs. (**E**) Co‐expression modules depicting mRNA and protein expression levels for all transcript–protein pairs.

Principal component analysis demonstrated a highly similar pattern in relatedness between B cell subsets for transcriptomics and proteomics. Naive B cells separated from IgMD^+^ and IgM^+^ MBCs and were distinct from switched MBCs (Figure 2A). The different types of switched memory B cell subsets showed limited separation in PC1, suggesting phenotypic resemblance of these subsets. The order of B cell subsets along PC1 partially resembled the order of isotypes found on the Ig locus, whereas PC2 confirmed the close relatedness of biological replicates (Figure S3B). IgG4 MBCs were most distinct from naive B cells and clustered at the opposite end of PC1 and also exhibited the highest number of significantly different mRNAs and proteins (Figure 2B; Figure S3A).

K‐means clustering of the differentially expressed mRNAs (#7302) and abundant proteins (#742) subdivided both datasets into four clusters with distinct expression profiles (Figure 2C,D). mRNA clusters C and D and protein clusters 1 and 2 are driven by a high or low expression of genes in naive B cells. Proteins upregulated in naive B cells (cluster 2) included B cell identity transcription factors (PAX5, BACH2, and IRF8) and BCR signaling proteins (CD79A/B, CD22, BLNK). Gene Ontology (GO) enrichment analysis indicated cluster D to comprise pathways involved in B cell activation (Tables S3, S4, and S7). Clusters A and B and 3 and 4 were driven by high and low expression of genes in IgG4 MBCs, further discussed below.

For most identified proteins, the corresponding mRNA was also identified (#3322 = 95%, Figure 2C). Of these, 955 were significantly differentially expressed between the nine B‐cell subsets on either the mRNA or protein level. A weighted correlation network analysis was performed to elucidate transcript–protein dynamics and identify modules (ME) of protein and RNA with correlating expression patterns (Figure 2E) [35, 40]. This resulted in 11 modules (MEs), ranging in size from 28 to 479 transcript–proteins pairs.

In the module containing the largest fractions (68%) of transcript–protein pairs (ME1, 2, and 3), mRNA and protein dynamics were in concordance. The remaining (32%) of transcript–protein pairs showed varying discordance between mRNA and protein dynamics (ME4–ME11). Most prominently, in ME8 and ME9, RNA expression dropped or increased while protein abundance remained similar. This may reflect proteins with a long half‐life or protein storage.

GO enrichment analysis was used to identify molecular functions describing the transcript–protein pair MEs. ME2 describes transcript–protein pairs involved in B cell activation, proliferation, and BCR signaling, and were highest expressed in naive B cells and lowest in IgG4 MBCs. For ME5 GO enrichment revealed as molecular function “MHC class II activity” and indeed contained transcript–protein pairs for genes concerning antigen presentation and the formation of immunological synapses, like CD79B, LAT2, and several HLA class II proteins. In summary, comprehensive proteome and transcriptome analysis of naive and MBC subsets in human peripheral blood revealed that mRNA and protein expression profiles separate B cell subsets according to differentiation status, correlated for many genes, and IgG4 MBCs were most distinct from naive B cells.

### 3.3 Common Signatures for Naive B Cells Versus MBCs

**FIGURE 3:**
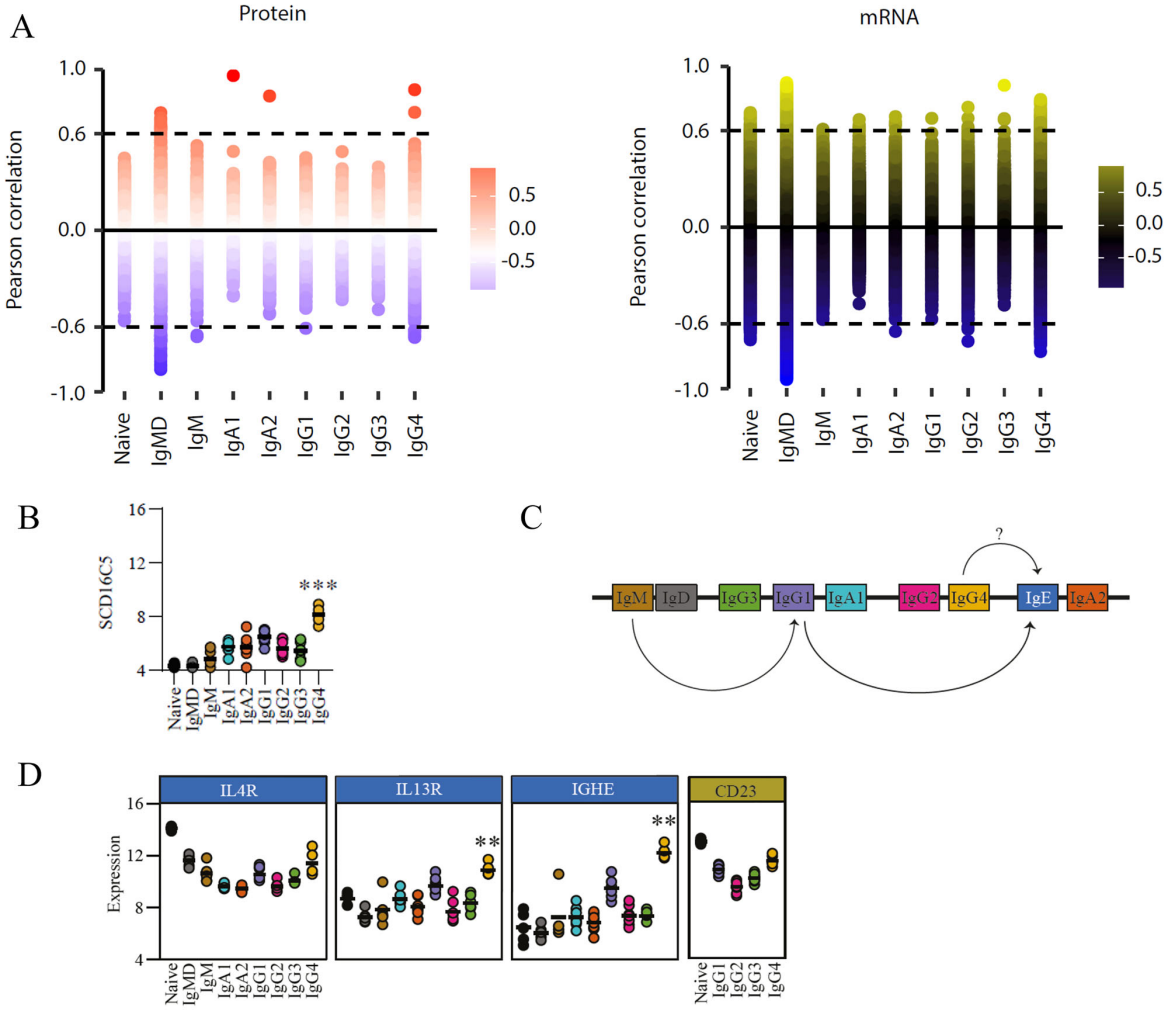
Protein/mRNA signature of IgG4‐swiched B cells. (**A**) B cell subset‐specific up‐ or downregulated mRNAs and proteins. Perfect fit analysis of mRNAs or proteins for each B cell subset (*p* <0.05). The dashed lines depict the cut‐off for upregulation >0.6 and cut‐off for downregulation <0.6 of mRNAs and proteins for a subset compared with all other B cell subsets. (**B**) mRNA expression levels of SDR16C5. (**C**) Schematic representation of the human IGH constant gene regions. Arrows indicate (potential [?]) sequential isotype switching events. (**D**) mRNA expression levels of IL4R, IL21R, IGHE, and CD23. (**B, D**) Bars represent medians. One‐way ANOVA and Tukey's multiple comparison test **p < 0.01, ***p < 0.001.

Prompted by the observation of clusters predominantly driven by up‐ or downregulation of mRNAs and proteins in naive B cells, we performed a supervised classification to identify mRNAs and proteins that follow this dynamic of an up or downregulation of mRNAs and proteins from naive B cells via IgMD MBCs, IgM MBCs, and switched MBCs, similar to what was observed for PC1 in the PCA (Figure 3A). Tables S5 and S6 show, for each B cell subset, upregulated mRNAs and proteins (>0.6) and downregulated mRNAs and proteins (←0.6). For all subsets, we identified corresponding IGH isotypes among upregulated mRNAs and proteins, which validates our approach. Note that we could not identify unique peptides for IGHG1, IGHG2, and IGHG3 due to high homology; therefore, no intensity value could be assigned to these proteins. For most subsets, we identified several specifically up‐ or downregulated mRNAs, but fewer proteins.

The common naive B cell protein signature consisted of 17 proteins with higher levels and 41 proteins with lower levels in naive B cells compared with MBCs (Table S6). This signature included surface receptors IGHD, CD22, and CD72, BCR‐associated lipid raft protein RFTN1, and AKT mediator TCL1A, all expressed in high abundance compared with MBCs, as well as signaling regulators Galectin‐1, DOK3, and THEMIS2, integrins ITGB1 and ITGAM, and myeloid cell nuclear differentiation antigen MNDA, all expressed at low abundance in naive B cells compared with MBCs. This naive B cell signature was also observed at the mRNA level, and further included high abundance of surface receptors including LAIR1, TLR1, FCRL1, FcεR, interleukin receptors IL4R, IL21R, and transcription factors BTLA, BCL6, BACH2, and FOXO1, and low abundance of surface receptors CD27, CD80, CXCR3, CD70, FAS, CD58, interleukin receptors IL2RA, IL6R, IL10R, IL15RA, and BAFF/APRIL receptor TNFRSF13B (Table S5). As expected, CD27 mRNA expression was absent in naive B cells and upregulated in MBC subsets. CD27 protein was not detected in any subset despite the CD27‐dependent cell‐sorting strategy for MBC subsets, indicating the difficulty to detect lower abundant cell‐surface proteins in bulk proteomics.

For IgMD MBCs, no proteins were found that were selectively highly expressed, but leukocyte immunoglobulin‐like receptor LILRA4 and retinoic acid receptor RXRA transcripts were highly abundant. For IgM, NLRP3 inflammasome transcripts were abundant. For IgA1 and IgA2, no specific highly abundant proteins were found, but IgA2 did express abundant mucosal homing CCR9 transcripts. Also, for IgG1, IgG2, and IgG3, no selectively abundantly expressed proteins were identified, but IgG2 MBCs differentially expressed, among others, cell adhesion mediator TGFBI, and IgG3 expressed metal transporter SLC11A1 and phospholipase PLCL1. It is also interesting to point out the slightly elevated mRNA expression of CXCR3 on IgG1/3, fitting a Th1 signature as described before (Figure S4) [41]. IgG4‐specific features are discussed below. Altogether, these data revealed that naive B cells have a unique signature and isotype‐defined MBCs share many of the same transcripts and proteins, but for several MBC subsets, specific upregulated transcripts and proteins could be identified.

### 3.4 Unique Phenotype of IgG4 MBCs

PCA analysis revealed that IgG4 MBCs were most distinct from naive B cells and less similar to other isotype‐defined MBCs. Furthermore, K‐means clustering analysis identified distinct mRNA and protein expression profiles for IgG4 MBCs: Cluster B (mRNA) and 3 (protein) define mRNA and proteins upregulated in IgG4 MBCs, whereas cluster A (mRNA) and 4 (protein) define downregulated mRNAs and proteins, respectively (Figure 2D).

Zooming in on specific mRNAs/protein expression in IgG4 MBCs, a number of selectively downregulated mRNAs that stand out include FYN and HCK, linked to tyrosine kinase activity (Table S5). Indeed, SYK is also found to be selectively downregulated (−0.57), and the downregulation of SYK was also identified on the protein level, confirming the diminished expression of this molecule for IgG4 MBCs relative to all other subsets (Table S6). Furthermore, mRNA levels of NF‐kB signaling‐associated TNFRSF18, CARD11, and EFHD2 are also downregulated. We also confirmed the previously identified downregulation of CXCR3 transcripts and CR2 for IgG4 compared with IgG1 MBCs (Figure S3, [23]). Furthermore, the most downregulated transcript in IgG4 MBCs compared with all other subsets was that of the neonatal Fc receptor (FCGRT/FcRn), important for IgG recycling. Another Fc receptor, FCGR2B, was not amongst the most highly downregulated mRNAs, but still selectively less expressed in IgG4 MBCs (−0.5).

Selective, highly upregulated mRNAs in IgG4 compared with all other subsets (Table S5) include LDLR, PAX5 repressor TLE4, and chemokine receptor CCR1. Furthermore, SDR16C5 mRNA is highly upregulated (Figure 3B), and this specific upregulation was confirmed at the protein level. SDR16C5, also known as RDH‐E2, functions as an oxidoreductase of all‐trans‐retinol and serves as a precursor for bioactive metabolites retinal and retinoic acid.

Further transcriptome analysis revealed an upregulation of IL‐4R on IgG4 MBCs versus all other IgG subsets and an IgG4‐specific upregulation of the IL‐13RA1 (Figure 3D). Cytokines corresponding to these receptors, IL‐4 and IL‐13, promote class switch toward IgG4 but also to IgE. Surprisingly, IgG4 MBCs display upregulated expression of IGHE transcripts, which was not detected on the protein level (Figure 3C,D). Also, tolerance‐associated cytokine IL‐10 may promote class switch toward IgG4. IL‐10RA expression levels were similar for IgG4^+^ MBCs compared with other MBC subsets, and IL‐10 was not detected for IgG4 MBCs on both mRNA and protein levels, suggesting that (resting) IgG4^+^ MBCs do not produce IL‐10 to promote tolerance in an autocrine or paracrine fashion. IL‐21R expression was similar for IgG4 MBCs compared with other MBC subsets but lower compared with naive and IgMD^+^ MBCs. IgG4 MBCs also showed higher expression of FcεRII (CD23) compared with other IgG MBC subsets but not compared with naive B cells (CD23, Figure 3D), suggesting increased capacity for IgG4^+^ MBCs to respond to IgE molecules.

Taken together, novel features for IgG4 MBCs are identified in this study, which suggest not only phenotypical differences, but also functional differences for IgG4 MBCs in peripheral blood.

## 4 Discussion

We generated proteomics and transcriptomics datasets of naive and isotype‐defined MBC subsets derived from human peripheral blood. Combined proteome and transcriptome analysis revealed that mRNA and protein expression profiles separate B cell subsets broadly according to isotype, in particular separating naïve from non‐naïve subsets. mRNA and protein expression levels correlated reasonably well for many genes. IgG4 MBCs were identified as being most distinct from naive B cells, which implies that there exist options for selective targeting of this B cell subset, to either enhance immune tolerance or counteract IgG4‐switched autoantibody production.

Combined proteome and transcriptome analysis reinforces confidence and allows for robust pathway analysis. These datasets provide a resource for the investigation of human naive and isotype‐defined MBC identity and function. Due to the low frequency of IgG4 MBCs, we could not further define MBC subsets using additional relevant phenotypic markers such as CD21, CD24, and CD95. In total, we identified 3511 proteins across all subsets, of which 738 proteins were differentially expressed, mostly between MBC subsets compared with naive B cells. Naive and memory B cells also exhibit similar expression profiles for many genes, as both cell types exist in a relatively quiescent state when not actively participating in an immune response. In this study, cells were sorted CD38^−^ indicating that they were not activated. These shared genes are linked to a low metabolic rate and the maintenance of a dormant state, enabling both naive and resting memory B cells to survive long‐term without the high metabolic demands associated with active antibody production.

Nonswitched MBCs are considered more likely to re‐enter the GC reaction upon secondary antigen exposure, whereas class‐switched MBCs are more likely to undergo rapid differentiation toward an antibody‐secreting cell type. We observed that nonswitched MBCs exhibit a gene expression profile more similar to naive cells, suggestive of functional plasticity for this subset and less extensive differentiation compared with class‐switched MBCs. Compared with switched MBCs, these subsets had higher expression of genes involved in activation, antigen presentation, and cytokine signaling, in line with a previous study [3]. In that study, the nonswitched MBC subset was defined as being IgM^+^, comprising heterogeneous and undefined IgD expression levels. In the current study, we separated nonswitched MBC into IgM+IgD^low^ and IgM^+^IgD^+^ MBCs. IgD expression levels are important to include, as IgM^+^IgD^+^ MBCs likely reside in the marginal zone compartment and are raised in a different microenvironment, shaping different functional properties attributed to differential gene expression.

It is to be expected that different isotype‐defined MBC subsets display distinct functional characteristics that influence their ability to expand, migrate, and differentiate following activation. Of interest are differences in expression of surface receptors and secreted factors that can modulate responses to antigen, influence their migration capacity, and interact with other cell types in the local environment. IgA2^+^ MBCs expressed higher levels of CCR9, which directs migration to the small intestine [39]. CXCR3, important for recruitment to inflammatory sites, was upregulated on all isotype‐switched MBCs, but less so for IgG4 B cells. The CXCR3 pathway plays a crucial role in the recruitment of inflammatory cells in settings of chronic inflammation, for example, in allergic and autoimmune diseases. This suggests less CXCR3‐mediated recruitment of IgG4 MBCs into inflamed sites. We did, however, observe upregulation of CCR1 on IgG4 MBCs compared with other MBC subsets, which may serve a similar function [42, 43].

Upon BCR cross‐linking, the intracellular domain (ITAM) of the Igα/Igβ heterodimer recruits protein tyrosine kinases (PTKs) for initiation and transduction of subsequent signaling events. This signaling cascade can promote different biological outcomes depending on the expressed BCR isotype and additional signals received by the B cell [44, 45, 46]. ITAM‐bound Syk can be phosphorylated, thereby lowering the threshold for activation, augmenting B cell survival, proliferation, and plasma cell differentiation [47, 48]. Intriguingly, IgG4 MBCs expressed lower levels of many PTKs, including, for example, Syk, Lyn, Fyn, and HCK, suggesting lowered BCR signaling, which may reflect the low frequency of IgG4 MBCs and limited IgG4 plasma cell generation.

Retinol metabolism is important in gut IgA MBCs and plays a role in tolerance. Retinol, or vitamin A, is an essential nutrient for a healthy immune system. Retinol serves as a precursor for bioactive metabolites retinal and retinoic acid, the latter of which is critical for B cell development, proliferation, and differentiation. Protein levels of SDR16C5 were upregulated in IgG4 B cells compared with all tested B cell subsets. SDR16C5 functions as an oxidoreductase of all‐trans‐retinol and serves as a precursor for bioactive metabolites retinal and retinoic acid. If the upregulated expression of SDR16C5 leads to enrichment of retinol‐derived bioactive metabolites, their function might play a role in the tolerance‐associated phenotype of IgG4 MBCs, similar to what is observed for IgA B cells at mucosal surfaces.

The capacity of MBCs to undergo isotype‐switching and selection is essential for understanding secondary immune responses. Previous studies that performed antibody repertoire analysis of peripheral blood B cells revealed that the majority of switch events occur from IgM to IgG1 and IgA1, and switching to IgG2, IgG4, IgE, and IgA2 may more often occur through indirect switching [49]. IgG4^+^ MBCs, and to a lesser extent IgG1 MBCs, displayed upregulation of IL‐13R, IL‐4R, and IGHE transcripts but not protein. Note that we performed bulk RNAseq and therefore cannot determine whether the majority of IgG4 MBCs express IGHE transcripts or just a fraction with very high expression levels. It is tempting to speculate that IgG4 MBCs, like IgG1 MBCs [49], may be able to isotype‐switch to IgE, which is located downstream of IgG4 on the IGH locus. A similar situation appears in mice, where switching from IgG1 to IgE may take place [50]. IgE antibodies show signs of affinity maturation, but on average have lower mutation levels as seen for IgG4 [51], making IgG1 and IgM more likely precursor candidates. Alternatively, these may also reflect putative sterile transcripts, observed during in vitro cultures of B cells upon addition of IL‐4, which promotes switch to IgE, that do not encode for functional IgE antibodies [52, 53, 54, 55]. We recently observed a profound inhibition of the IL‐4R blocking antibody dupilumab on IgG4 switching following repeated mRNA vaccination [56]. It is tempting to speculate a prominent role of sequential switching via IgG1 toward IgG4, which was interrupted at the B cell level via IL‐4R inhibition. In contrast to IgG4 switching, IgG1 switching in vitro is still observed in the absence of IL‐4 [25].

In summary, IgG4 MBCs share many features with other MBC subsets, but differential expression of RTKs, STATs, and IL and FcγR surface receptors by IgG4 MBCs indicates additional specialization based on isotype. However, functional studies are necessary to determine the functional consequences. These findings provide insight into IgG4 MBC biology and will enable further studies to interrogate IgG4 MBCs in the context of allergy and IgG4‐mediated autoimmune disease. Collectively, combined transcriptomics and proteomics revealed isotype‐defined phenotypic specialization of human MBCs and, in particular, identified a distinct profile for IgG4‐switched B cells.

## 5 Data Limitations and Perspectives

This study reports a detailed evaluation of the transcriptomic/proteomic signatures of isotype‐specific human B cell subsets. Multiple characteristic markers were found for which the proteomics dataset confirmed the transcriptomic findings and vice versa. Therefore, we did not include additional validations by, for example, FACS analysis; both data sets serve as each other's validation.

## Author Contributions

**Jana Koers**: Conceptualization, investigation, analysis, writing, original draft, writing, review, and editing. **Arie J. Hoogendijk**: Analysis, writing, review, and editing. **Simon Tol**: Investigation, writing, review, and editing. **Floris P. J. Van Alphen**: Investigation, writing, review, and editing. **Ninotska I.L. Derksen**: Investigation, writing, review, and editing. **Maartje van den Biggelaar**: Conceptualization, supervision, writing, review, and editing. **Theo Rispens**: Conceptualization, supervision, writing, review, and editing.

## Conflicts of Interest

The authors declare no conflicts of interest.

## Supporting information




**Supporting File 1**: eji70159‐sup‐0001‐TableS1.xlsx.

**Supporting File 2**: eji70159‐sup‐0002‐TableS2.xlsx.

**Supporting File 3**: eji70159‐sup‐0003‐TableS3.xlsx.

**Supporting File 4**: eji70159‐sup‐0004‐TableS4.xlsx.

**Supporting File 5**: eji70159‐sup‐0005‐TableS5.xlsx.

**Supporting File 6**: eji70159‐sup‐0006‐TableS6.xlsx.

**Supporting File 7**: eji70159‐sup‐0007‐TableS7.xlsx.

**Supporting File 8**: eji70159‐sup‐0008‐Figures.pdf.

## Data Availability

The data supporting the findings of this study are openly available: the mass spectrometry data have been deposited to the ProteomeXchange Consortium (http://proteomecentral.proteomexchange.org) via the PRIDE partner repository with dataset identifier PXD060953. RNAseq data are deposited in the GEO repository (https://www.ncbi.nlm.nih.gov/geo/) and can be accessed with accession number GSE298777.
