# Supplementary material for: Transcriptome–Proteome Analysis of Human Naive and Memory B Cell Subsets Reveals Isotype and Subclass‐Specific Phenotypes
Source: Eur J Immunol. 2026 Mar 12;56(3):e70159. doi: 10.1002/eji.70159 (PMC12981214; doi:10.1002/eji.70159)
Supplement: Supplementary file 8 — Supporting File 8: eji70159‐sup‐0008‐Figures.pdf. [file EJI-56-e70159-s006.pdf]

A

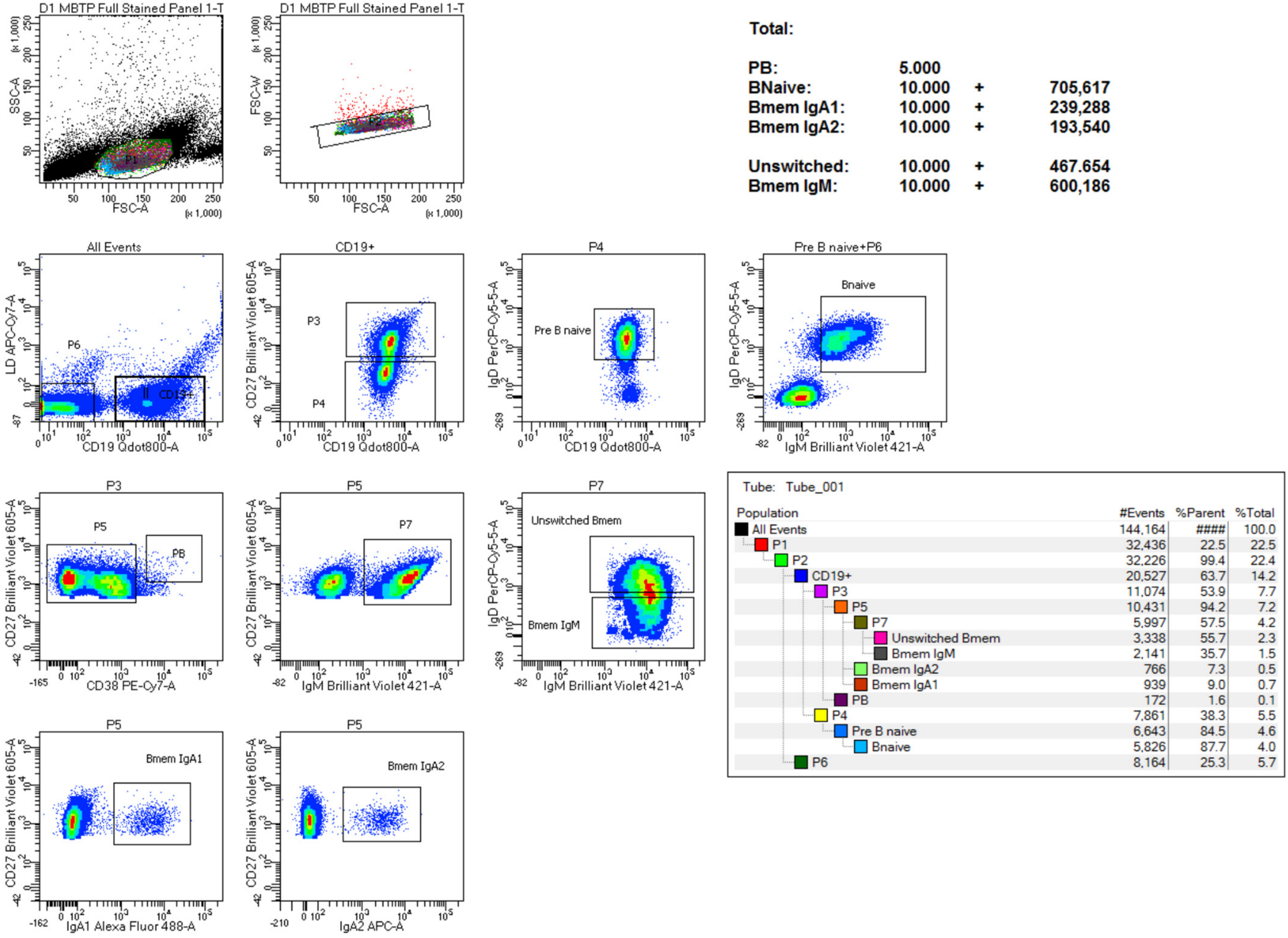

Total:

**PB:**	**5.000**		
**BNaive:**	**10.000**	**+**	**705,617**
**Bmem IgA1:**	**10.000**	**+**	**239,288**
**Bmem IgA2:**	**10.000**	**+**	**193,540**
**Unswitched:**	**10.000**	**+**	**467.654**
**Bmem IgM:**	**10.000**	**+**	**600,186**

Tube: Tube_001

Population	#Events	%Parent	%Total
All Events	144,164	####	100.0
P1	32,436	22.5	22.5
P2	32,226	99.4	22.4
CD19+	20,527	63.7	14.2
P3	11,074	53.9	7.7
P5	10,431	94.2	7.2
P7	5,997	57.5	4.2
Unswitched Bmem	3,338	55.7	2.3
Bmem IgM	2,141	35.7	1.5
Bmem IgA2	766	7.3	0.5
Bmem IgA1	939	9.0	0.7
PB	172	1.6	0.1
P4	7,861	38.3	5.5
Pre B naive	6,643	84.5	4.6
Bnaive	5,826	87.7	4.0
P6	8,164	25.3	5.7

Figure continues on next page

B

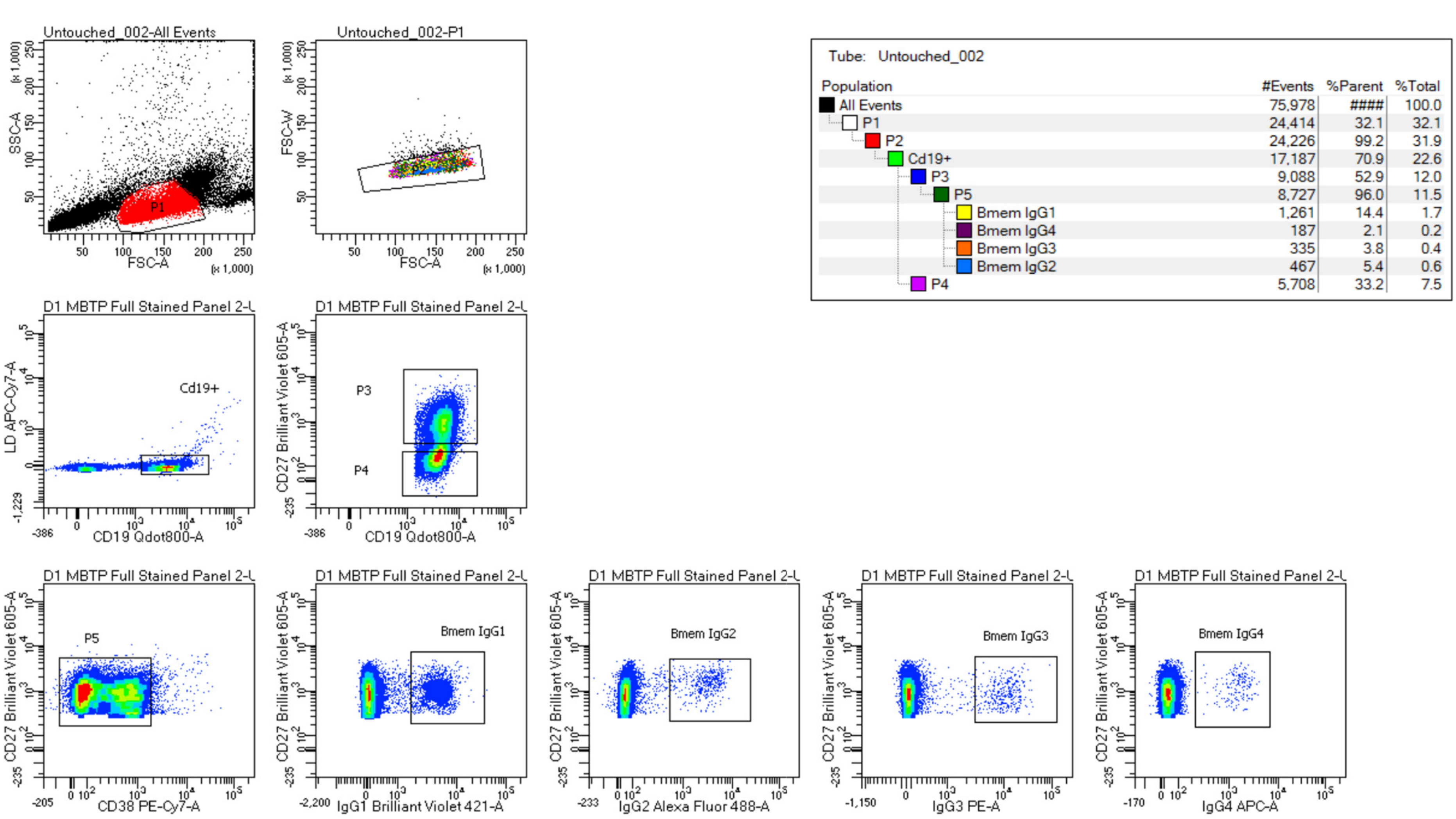

Population	#Events	%Parent	%Total
All Events	75,978	####	100.0
P1	24,414	32.1	32.1
P2	24,226	99.2	31.9
Cd19+	17,187	70.9	22.6
P3	9,088	52.9	12.0
P5	8,727	96.0	11.5
Bmem IgG1	1,261	14.4	1.7
Bmem IgG4	187	2.1	0.2
Bmem IgG3	335	3.8	0.4
Bmem IgG2	467	5.4	0.6
P4	5,708	33.2	7.5

Total: **93.401.235**

IgG4: **10.000 +** **125.629**
IgG1: **10.000 +** **930,935**
IgG2: **10.000 +** **300,343**
IgG3: **10.000 +** **246,211**

Figure continues on next page

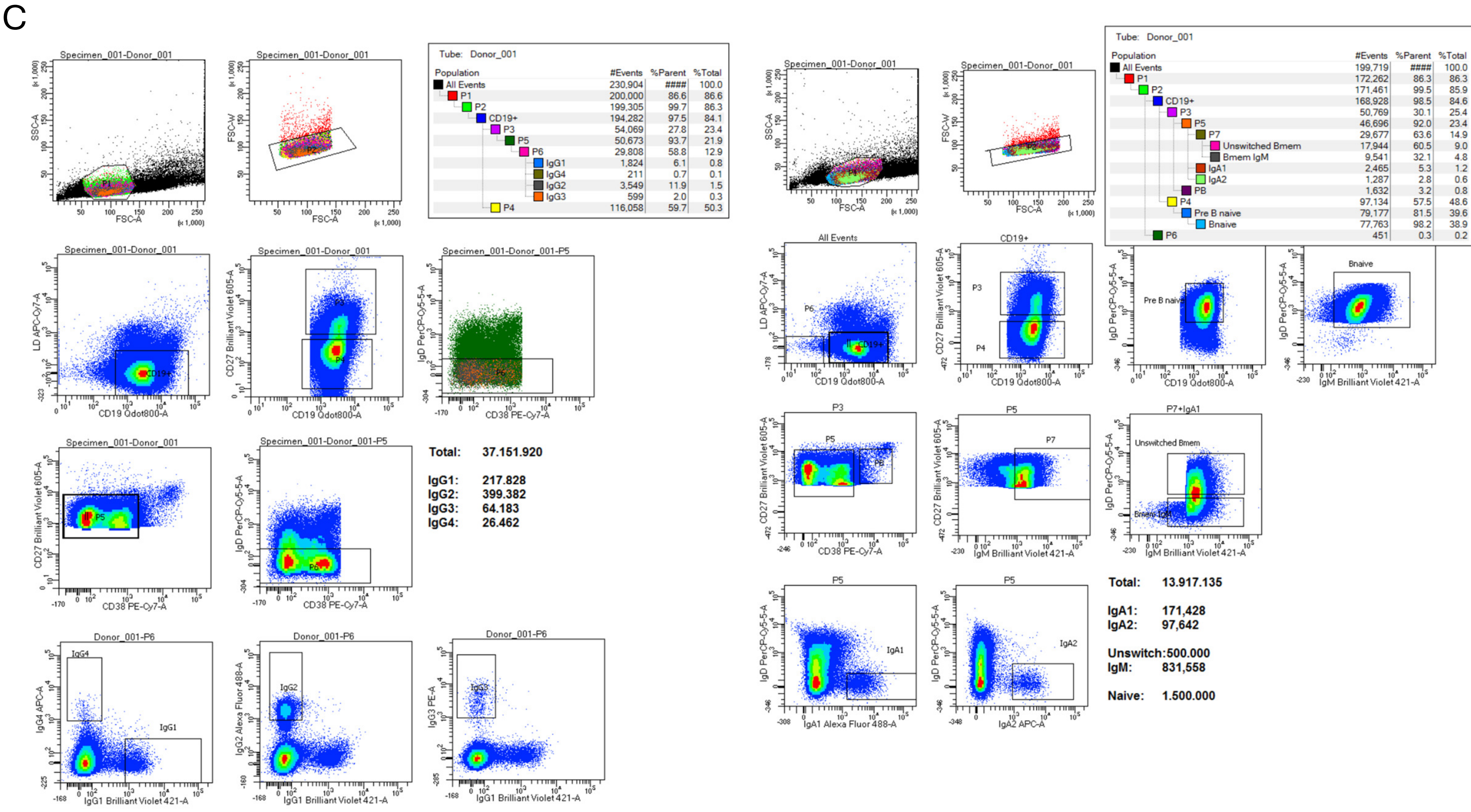

Figure S1. Examples of gating strategies for sorting of the different subsets; A-B: for proteomics analysis, C: for transcriptomics analysis.

Supplementary materials

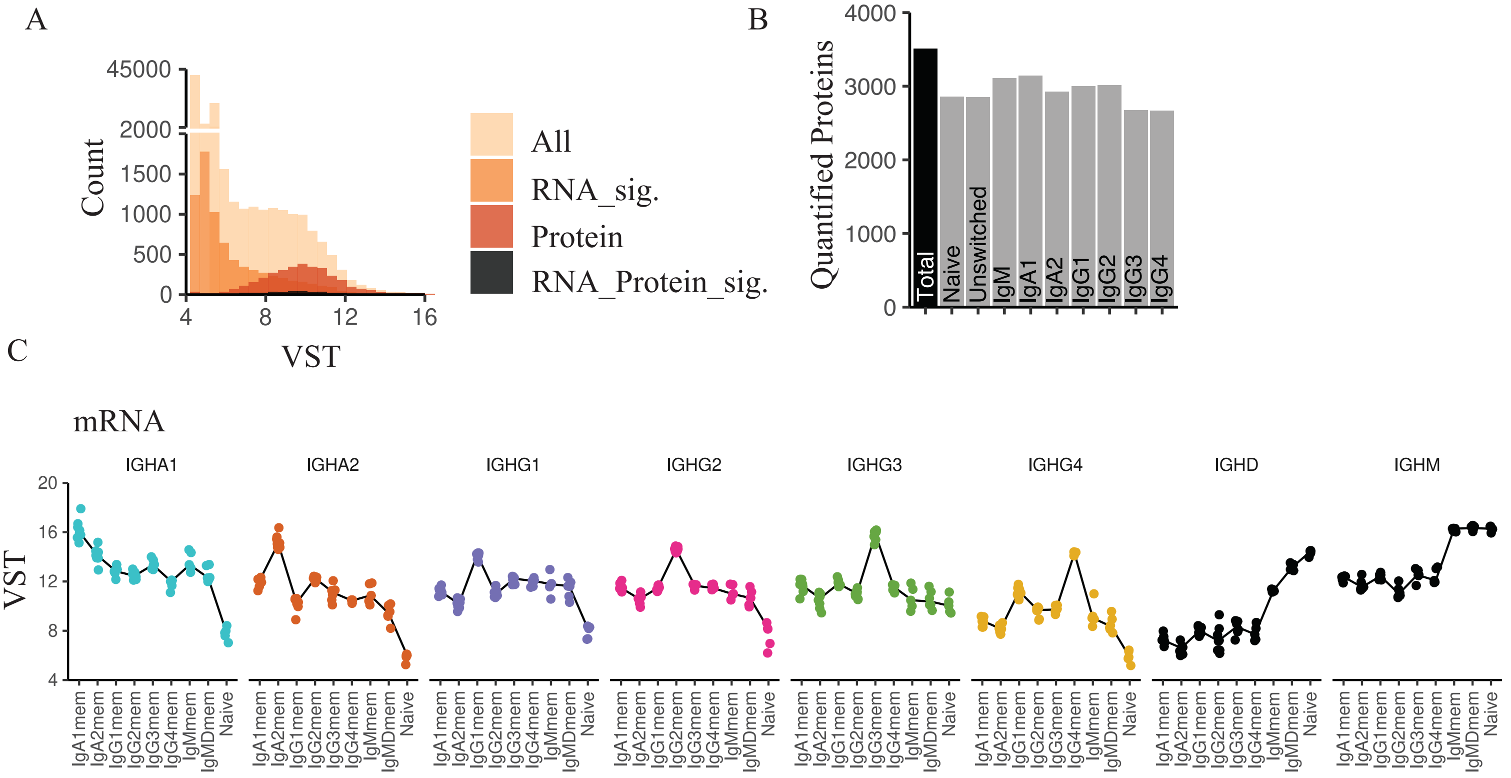

Figure S2. RNAseq and protein level comparison. (**A**) Hisogram of RNA expression range depicted as VST. (**B**) Number of quantified proteins for each of the B cell subsets. (**C**) mRNA expression levels for each IGH gene in each B cell subset.

A

	Down	Up
NaiveBcells - BmemUnswitched	28	15
NaiveBcells - Bmem_IgM	85	47
NaiveBcells - Bmem_IgA1	92	54
NaiveBcells - Bmem_IgA2	137	112
NaiveBcells - Bmem_IgG1	102	102
NaiveBcells - Bmem_IgG2	115	76
NaiveBcells - Bmem_IgG3	106	69
NaiveBcells - Bmem_IgG4	152	180
BmemUnswitched - Bmem_IgM	2	4
Bmem_IgA1 - Bmem_IgA2	1	1
Bmem_IgA1 - Bmem_IgG1	0	3
Bmem_IgA1 - Bmem_IgG2	1	1
Bmem_IgA1 - Bmem_IgG3	0	2
Bmem_IgA1 - Bmem_IgG4	10	54
Bmem_IgA2 - Bmem_IgG1	16	49
Bmem_IgA2 - Bmem_IgG2	0	1
Bmem_IgA2 - Bmem_IgG3	2	2
Bmem_IgA2 - Bmem_IgG4	8	31
Bmem_IgG1 - Bmem_IgG2	2	1
Bmem_IgG1 - Bmem_IgG3	3	4
Bmem_IgG1 - Bmem_IgG4	53	85
Bmem_IgG2 - Bmem_IgG3	0	0
Bmem_IgG2 - Bmem_IgG4	11	34
Bmem_IgG3 - Bmem_IgG4	7	28

B

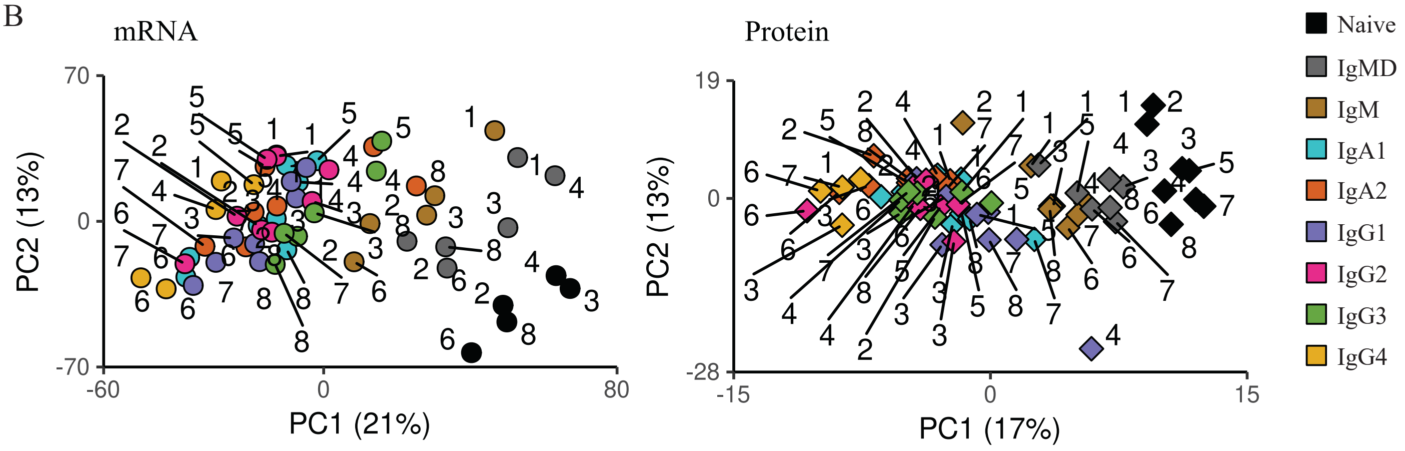

Figure S3. (**A**) Pairwise comparison of differentially expressed proteins between all subsets. (**B**) PCA analysis as in Fig. 2A with depiction of donor number per data point.

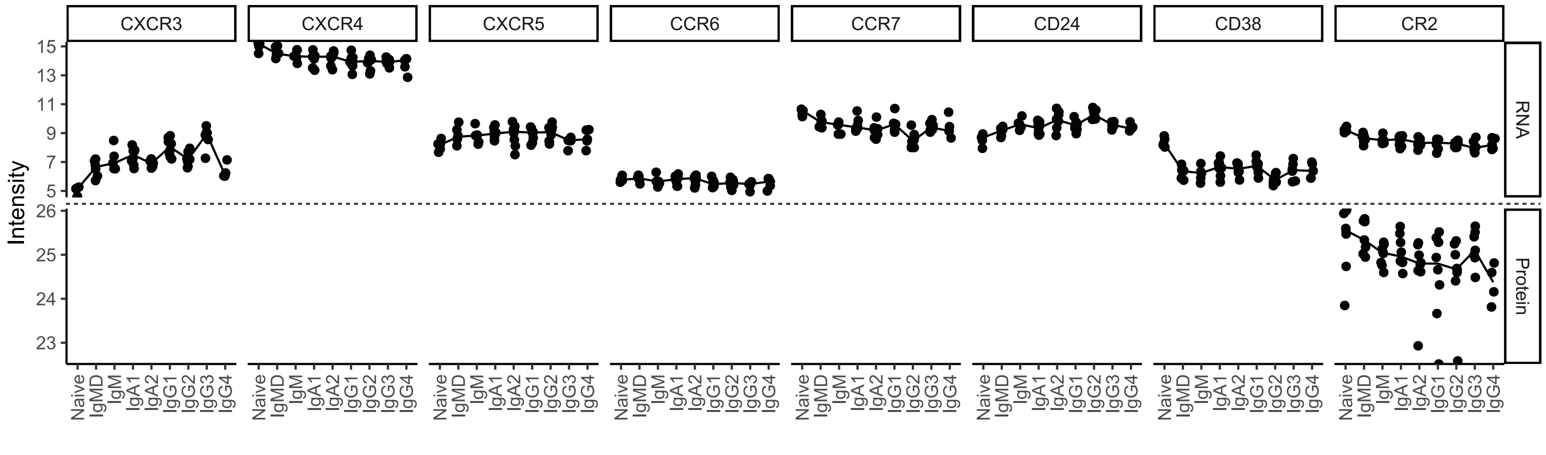

Figure S4. mRNA and protein expression levels of previously identified proteins differentially expressed between IgG1 and IgG4 B cells. For many targets no protein was detected.

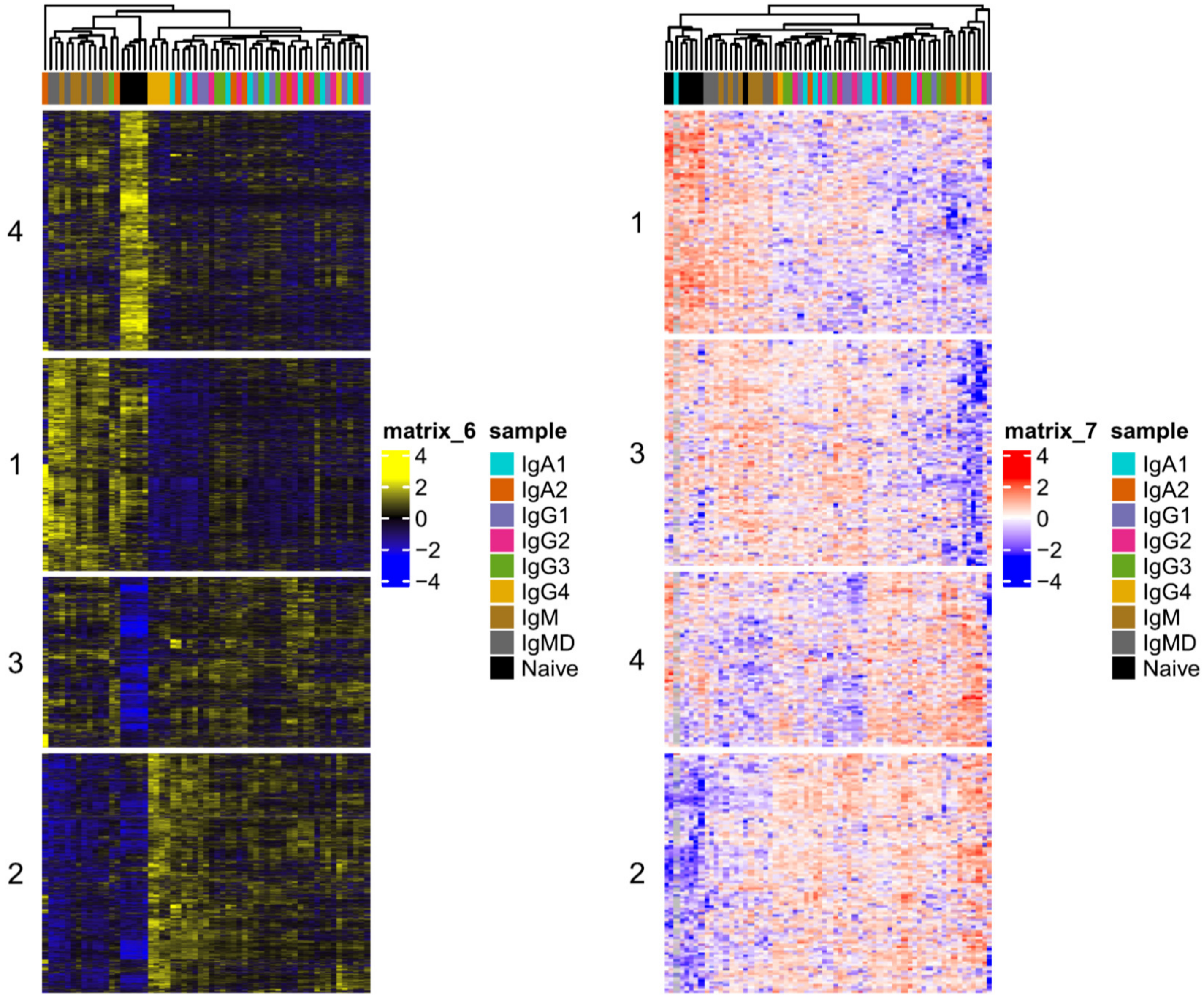

Figure S5. Transcriptomics and proteomics profiles of B cell subsets. Heatmaps display the number of significantly differently expressed mRNAs (left) or proteins (right), based on hierarchical clustering.
